# Prevalence and correlates of severe under-5 child anthropometric failure measured by the composite index of severe anthropometric failure in Bangladesh

**DOI:** 10.3389/fped.2022.978568

**Published:** 2022-09-14

**Authors:** Mohammad Rocky Khan Chowdhury, Hafiz T. A. Khan, Mamunur Rashid, Md. Nazrul Islam Mondal, Farzana Akhter Bornee, Baki Billah

**Affiliations:** ^1^Epidemiology and Preventive Medicine, School of Public Health and Preventive Medicine, Faculty of Medicine, Nursing and Health Sciences, Monash University, Melbourne, VIC, Australia; ^2^Health Promotion and Public Health, College of Nursing, Midwifery and Healthcare, University of West London, London, United Kingdom; ^3^Department of Public Health Science, Faculty of Occupational and Health Sciences, University of Gävle, Gävle, Sweden; ^4^Population Science and Human Resource Development, University of Rajshahi, Rajshahi, Bangladesh; ^5^Department of Pediatrics, Bangabandhu Sheikh Mujib Medical University, Dhaka, Bangladesh

**Keywords:** Bangladesh, children, severe malnutrition, determinants, undernutrition

## Abstract

**Background:**

Although Bangladesh has made noticeable progress in reducing the prevalence of stunting, wasting, and being underweight among under-5 children, it has not been very successful in reducing overall severe anthropometric failure (SAF) among them. Therefore, the study aims to identify the prevalence and risk factors of SAF measured by the Composite Index of Severe Anthropometric Failure (CISAF) among under-5 children in Bangladesh.

**Methods:**

Data was drawn from a cross-sectional Bangladesh Demographic Health Survey (BDHS) conducted in 2017–2018. A bivariate analysis (Chi-square test) and logistic regression analysis were used to estimate the unadjusted, and age and sex-adjusted prevalence of SAF. Odds ratio (OR) and confidence interval (CI) were assessed using logistic regression analysis to identify the various risk factor of SAF.

**Results:**

The overall adjusted prevalence of under-5 child SAF was 11.3% (95% CI: 10.6–12.0) and it was highly prevalent among children of uneducated mothers (adjusted, 22%, 95% CI: 17.3–26.8). The key factors associated with SAF were children in the age group 24–35 months (OR: 2.43, 95% CI: 1.83–3.23), children born with low birth weight (OR: 3.14, 95% CI: 2.24–4.97), children of underweight mothers (OR: 1.82, 95% CI: 1.44–2.29), children of parents with no formal education (OR: 2.28, 95% CI: 1.56–3.31) and children from lower socio-economic status (OR: 2.25, 95% CI: 1.55–3.26).

**Conclusion:**

Prioritizing and ensuring context-specific interventions addressing individual, community, public policy, and environment level risk factors from policy level to implementation to reduce structural and intermediary determinants of under-5 SAF.

## Introduction

Anthropometric failure (AF) in children shows itself in the form of stunting, wasting, and being underweight and remains one of the biggest health problems in low- and middle-income countries, including Bangladesh (1). Globally, around 195 million children under-5 suffer from some form of AF (2). Further, ~3 million children under-5 die from AF (2). Reduced dietary intakes of macro- and/or micronutrients and increased losses or enhanced requirements are some established causes of AF among under-5 children (3). Children with AF are physically, emotionally, and intellectually less productive than well-nourished children due to incompatible growth and development (4). In addition, short and long-term adverse health consequences including a degree of cognitive impairment, weak thymus development, a decreased peripheral lymphocyte count, an increased susceptibility to infections, and a poor immune system are all directly related to the severity of AF and often lead to death of a child have multiple exposures of these health consequences (5). Nearly 20 million children globally suffer from severe wasting (6), resulting in 1 million deaths per year by increasing susceptibility to death from severe infection (7). Children having severe wasting are usually 12 times more likely to die than a non-wasted or stunted child. On the other hand, a child having severe stunting also carries five times high risk of death than a non-wasted or stunted child (8).

Bangladesh has been identified as having a high burden of AF in its under-5 with a more than 40% prevalence rate (9). Although the country has made significant improvements in addressing the Millennium Development Goals (MDG) 1 (eradication of extreme poverty and hunger) and MDG-4 (child mortality), it has not been very successful in reducing AF in children particularly in its severest form (10). In Bangladesh, around 20% of children with severe anthropometric failure (SAF) were identified in 2014 (9). Further, factors, including poor maternal education, poor household economic status, low birth weight, poor infant and young child feeding practices, frequent infections, and inadequate access to health care, unsafe water and sanitation complexly interacted with severe stunting, severe wasting and being severe underweight in low and middle income countries including Bangladesh (9, 11, 12). These studies, however, only identified the risk factors for single form or multiple concurrent forms of severe stunting, severe wasting and being severely underweight that partly overlap, thus not providing a convincing estimate of the proportion of AF among children in the population.

The Composite Index of Anthropometric Failure (CIAF) uses conventional nutritional and growth indicators (stunting, wasting and underweight) to provide six different measurements, and the overall burden of AF was estimated by aggregating the values of conventional nutritional and growth indicators (13). The CIAF therefore, widely estimate the proportion of children with AF in the population (13). The Composite Index of Severe Anthropometric Failure (CISAF) followed by the methodological approach of CIAF that provides a comprehensive view of the overall burden of SAF in poor-resource settings (14). However, in Bangladesh, some socio-demographic factors were identified as risk factors of AF measured by the CIAF (15), no previous study used the CISAF to explore the national prevalence and complex interplay between individual, community, public policy and environment level risk factors and under-5 child SAF in Bangladesh. However, this study has considered already known etiology to identify the associated factors of SAF, investigating the change of direction of these factors using more recent data, especially in the context of SAF as per the CISAF, might be helpful for revising important policy decision-making. Therefore, we aim to explore the prevalence and risk factors of SAF by the CISAF in Bangladesh to help provide important insights for developing appropriate policy initiatives to address the severe adverse nutritional and growth outcome of under-5 children in the country.

## Methods

### Data source and study design

**Figure 1:**
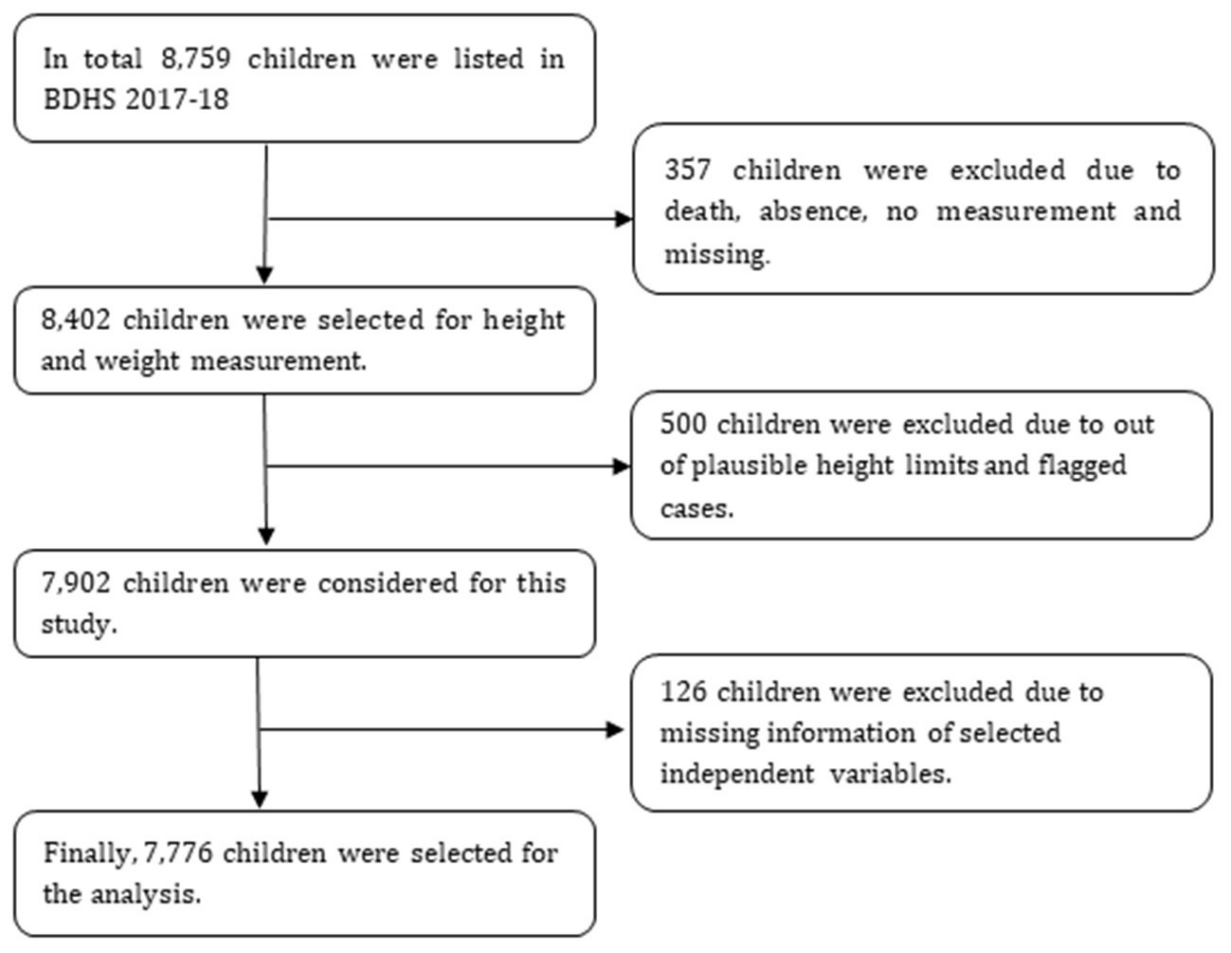
Schematic presentation of sample size selection.

This study used data drawn from the 2017–2018 Bangladesh Demographic Health Surveys (BDHS) that showing around 99% response rate. In BDHS 2017–2018, enumeration areas (EAs) or primary sampling units (PSUs) and households were selected using multistage stratified sampling techniques to collect data from of adults (male and female) and children residing in non-institutional dwellings. The data collection was started on 24 October 2017 and ended on 15 March 2018. At the first stage, 675 PSUs (250 PSU from urban and 425 PSU from rural) were randomly selected from 293,579 PSUs (clusters) of the last census survey 2011 designed by the Bangladesh Bureau of Statistics using probability proportional to size technique. At the second stage, a systematic sample of 30 households from each PSU were selected using an equal probability systematic sampling technique. This multistage sampling technique, including its sampling weight, helps reduce potential sampling bias. In addition, all ever-married women aged 15–49 years from the pre-selected households were interviewed without replacement and change in the implementing stage to prevent selection bias (16). Informed consent was obtained verbally from each participant to collect information about them and their children before enrolling in the study. A significant number of the study sample was illiterate, so verbal consent was considered the most suitable option to confirm participation (16). The BDHS samples include socio-demographic data, health and nutritional indicators from Bangladeshi adults using a standard questionnaire. In 2017-18 survey, 8,759 under-5 children were found eligible for anthropometric measurement of which 7,776 were found credible for analysis (Figure 1). Further details of BDHS 2017-18 can be found elsewhere (16).

### Outcome variable

**Figure 2:**
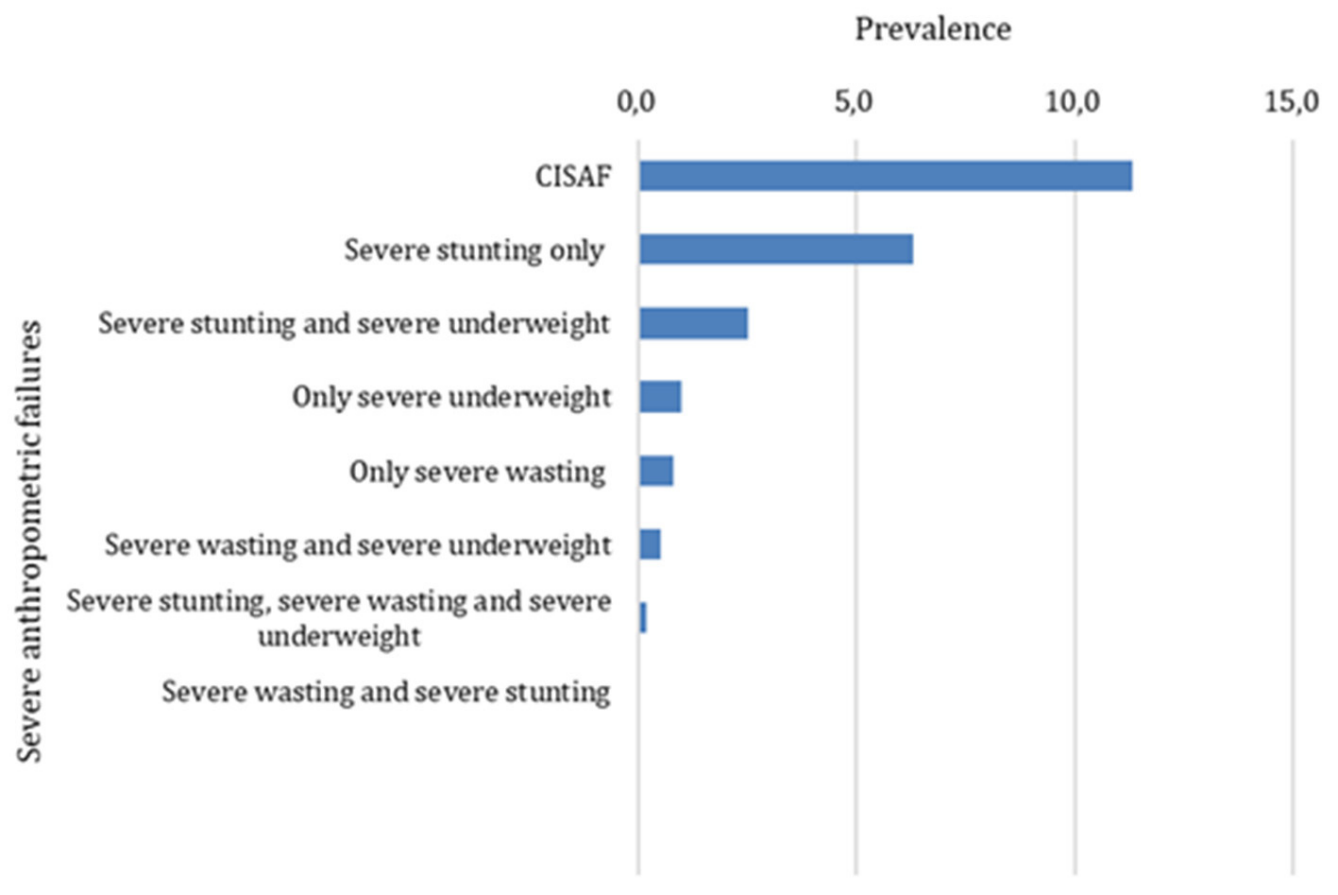
Prevalence of under-5 SAF.

Severe nutritional and growth indicators for under-5 children were categorized into seven groups: (A) no severe failure; (B) severe wasting only; (C) severe stunting only; (D) severe underweight only; (E) severe wasting and severe underweight; (F) severe stunting and severe underweight; (G) severe wasting and severe stunting; (H) severe wasting, severe stunting and severe underweight (Table 1). A child is considered to have SAF if she/he has a severe form of any one out of three SAF's indicators (B, C and D) or their combinations from E to H (Figure 2).

**Table 1:** Classification of children with severe anthropometric failure.

**Group name**	**Description**	**Severe wasting**	**Severe stunting**	**Severe underweight**
A	No severe failure	No	No	No
B	Severe wasting only	Yes	No	No
C	Severe stunting only	No	Yes	No
D	Severe underweight only	No	No	Yes
E	Severe wasting and severe underweight	Yes	No	Yes
F	Severe stunting and severe underweight	No	Yes	Yes
G	Severe wasting and severe stunting	Yes	Yes	No
H	Severe wasting, severe stunting and severe underweight	Yes	Yes	Yes

The primary outcome measure was under-5 SAF using the CISAF (14). A child was considered to be, respectively, severely stunted (stature too short for age), severely wasted (extremely thin) and severely underweight (very low weight for age) if the height-for-age, weight-for-height, and weight-for-age indices were three Standard Deviations (SDs) or more below the respective median of the World Health Organization (WHO) reference population (17).

### Independent variables

The literature indicates that a number of independent variables are important, and this study uses these variables to aid further investigation (9, 18, 19). The selected variables include maternal characteristics such as education of parents (both parents uneducated, only father uneducated—when mother educated, only mother uneducated—when father educated, both parents educated), mother's income-earning activities (currently not working, currently working), mother's BMI (≥ 18.5 kg/m^2^, <18.5 kg/m^2^), mothers received antenatal care (no, yes), mothers received postnatal care (no, yes), mother's attitude toward wife-beating (not justified, justified), mother's decision-making autonomy (not practiced, practiced) and number of living children (<3 children, three children and above); reproductive health related factors included unwanted child (no, yes), place of delivery (non-institutional, institutional), ever had terminated pregnancy (no, yes), last birth a cesarean section (no, yes) and sign of pregnancy complication (no, yes); children characteristics were children's age in months (0–11, 12–23, 24–35, 36–47, 48–59), sex of child (male, female), birth order (first, second third, fourth and above), low birth weight (no, yes) and currently had illness (no, yes); environmental characteristics included source of drinking water (improved, unimproved), solid fuel used in cooking (clean, solid) and type of toilet facility (improved, unimproved); household characteristics included mass media exposure through television, radio and newspaper/magazine (no, yes), and wealth index (poorest, poorer, middle, richer, richest); and contextual factor was place of residence (urban, rural) (16). Details explanations were provided in Appendix 1.

### Statistical analysis

The background characteristics of the children were calculated using descriptive statistics. A Bivariate analysis and logistic regression were used for assessing the unadjusted and adjusted prevalence of SAF based on the CISAF. In all analyses, the significance level was set at *p* < 0.25 (Chi-square test) to include variables found important (20–22). Adjusted model was developed to analyze the appropriate binary value for SAF as per the CISAF. All independent variables found to be significant in the bivariate analysis (Chi-square test) were simultaneously entered into the multiple regression models for adjustment. Odds ratio (OR), risk difference (RD) and confidence interval (CI) were assessed to identify the factors associated with SAF where *p* < 0.05. The most influential factors were assessed using Wald statistics. The data were analyzed by controlling cluster (PSU) and stratum (urban area: city corporation area and other than city corporation area and rural area) with sampling weights that represent the whole country, both urban and rural areas, to ensure the precision of the estimates. Multicollinearity was checked by examining the standard errors (SEs) of regression coefficients in the logistic regression analyses. An SE >2.0 indicates multicollinearity among the independent variables (23). The SEs for the independent variables in the adjusted models for each outcome were <1, indicating an absence of multicollinearity. Stata version 14.2 (StataCorp LP, College Station, Texas) was used for all analyses to take account of the complex nature of the sampling weight of the BDHS 2017-18. To adjust sampling weight in the analysis, the Stata command “svyset” was used.

## Results

Most of the women (around 35.0%) were from the age group 20–24 years. Mothers of ~7.0% of children had no formal education (both parents uneducated: 3.9% and only mothers were uneducated when fathers were educated: 3.2), fathers of around 11.3% of children had no formal education when mothers were educated, around 8% of mothers did not receive antenatal care and around 50% of birth delivery was non-institutional. More than two fifth (42.0%) of the children were <2 years of age and 52.0% of these were male. Around 42.0% of children were from low socio-economically and 68.0% of them were from rural areas (Table 2).

**Table 2:** Background characteristics of the respondents.

**Characteristics**	**Number**	**Percentage (%)**
Total	7,776	100.0
**Women's age (in years)**		
15–19	1,010	13.0
20–24	2,701	34.7
25–29	2,180	28.0
30–35	1,298	16.7
35 and above	587	7.6
**Parents' education**		
Both parents uneducated	295	3.9
Only father uneducated	862	11.3
Only mother uneducated	244	3.2
Both parents educated	6,257	81.6
**Mother income-earning status**		
Currently not working	4,617	59.4
Currently working	3,159	40.6
**Mother's BMI^a^**		
≥18.5 kg/m^2^	6,685	86.0
<18.5 kg/m^2^	1,090	14.0
**Mothers received antenatal care (*****n*** **= 4,610)**		
No	367	8.0
Yes	4,243	92.0
**Mothers received postnatal care (*****n*** **= 4,604)**		
No	1,598	34.7
Yes	3,006	65.3
**Mothers attitude toward wife-beating^b^**		
Not justified	6,320	81.3
Justified	1,456	18.7
**Mother's decision-making autonomy^c^**		
Practiced	1,066	13.9
Not practiced	6,591	86.1
**Number of living children**		
Less than 3 children	5,549	71.4
3 children and above	2,226	28.6
**Unwanted child**		
No	4,944	91.1
Yes	484	8.9
**Place of delivery**		
Non-institutional	2,416	50.3
Institutional	2,392	49.7
**Ever had terminated pregnancy**		
No	6,417	82.5
Yes	1,359	17.5
**Last birth a cesarean section**		
No	3,684	68.0
Yes	1,737	32.0
**Sign of pregnancy complication**		
No	2,752	64.9
Yes	1,491	35.1
**Children age**		
0–11 months	1,673	21.5
12–23 months	1,616	20.8
24–35 months	1,517	19.5
36–47 months	1,462	18.8
48–59 months	1,508	19.4
**Sex of child**		
Male	4,057	52.2
Female	3,719	47.8
**Birth order**		
First	2,973	38.2
Second	2,519	32.4
Third	1,321	17.0
Fourth and above	963	12.4
**Low birth weight (*****n*** **= 4,807)^d^**		
No	1,837	38.2
Yes	339	7.1
Not weighted	2,631	54.7
**Currently had illness^e^**		
No	4,039	52.0
Yes	3,732	48.0
**Source of water**		
Improved	6,764	87.0
Unimproved	1,012	13.0
**Solid waste use in cooking**		
No	2,294	29.5
Yes	5,475	70.5
**Type of toilet facility**		
Improved	4,427	56.9
Unimproved	3,349	43.1
**Maas media exposure^f^**		
No	2,722	35.0
Yes	5,054	65.0
**Wealth index^h^**		
Poorest	1,689	21.7
Poorer	1,589	20.4
Middle	1,492	19.2
Richer	1,569	20.2
Richest	1,436	18.5
**Place of residence**		
Urban	2,051	26.4
Rural	5,725	73.6

a Underweight measured as <18.5 kg/m^2^.

b A wife being beaten if she went out without telling her partner/ neglected the children/argued with her partner/burnt food/ was forced to have sex regardless of her consent.

c A woman usually can decide by herself or jointly on her healthcare/large household purchases/visits to family or relatives.

d Child's weight at birth measured as ≤ 2.5 kg.

e Child had at least one morbid condition out of diarrhea, fever and cough in the 2 weeks preceding the survey.

f Exposed television/radio/newspaper/magazine to some extent.

g Integrating household asset ownership and access to drinking water and sanitation.

### Prevalence of SAF based on the CISAF

The prevalence of overall severe stunting, severe wasting and severe underweight were, respectively, 9.1%, 4.2% and 1.8% (see Supplementary Figure 1). The adjusted prevalence of under-5 SAF based on the CISAF was 11.3%. The prevalence of only single form (only severe stunting, severe wasting or severe underweight) of SAF was 7.9% and multiple concurrent forms (severe stunting and severe underweight, severe wasting and severe underweight, and severe stunting, severe wasting and severe underweight) of SAF was (3.4%) (Figure 1). The adjusted prevalence of SAF was significantly higher consecutively among children of parents with (22.0%), children born with low birth weight (~19.0%), mothers that did not receive antenatal care (18.0%), children of underweight mothers (17.0%), children in fourth and above birth order (17.0%) and among children in the poorest section of the society (16.0%) (Table 3).

**Table 3:** Prevalence of SAF based on the CISAF among children under-5.

**Characteristics**	**Number**	**Unadjusted prevalence (95% CI)**	**Adjusted ^a^ prevalence (95% CI)**	***p* values (Chi-square)**
**Women's age (in years)**				
15–19	126	12.2 (10.1–14.6)	13.0 (10.9–15.2)	0.685
20–24	285	10.3 (9.0–11.9)	10.5 (9.3–11.6)	
25–29	248	11.2 (9.6–12.9)	11.3 (10.0–12.7)	
30–35	147	11.4 (9.3–13.8)	11.2 (9.5–12.9)	
35 and above	78	11.4 (8.9–14.3)	12.6 (10.0–15.2)	
**Parents' education**				
Both parents uneducated	65	22.5 (17.5–28.5)	22.0 (17.3–26.8)	<0.001
Only father uneducated	141	16.3 (13.5–19.4)	16.3 (13.8–18.7)	
Only mother uneducated	46	18.6 (13.7–24.7)	18.9 (13.9–23.8)	
Both parents educated	619	9.5 (8.6–10.5)	9.9 (9.1–10.6)	
**Mother income-earning status**				
Currently not working	499	10.2 (9.1–11.5)	10.8 (9.9–11.7)	0.018
Currently working	385	12.3 (11.0–13.7)	12.1 (11.0–13.3)	
**Mother's BMI**				
≥18.5 kg/m^2^	686	10.2 (9.2–11.3)	10.3 (9.6–11.1)	<0.001
<18.5 kg/m^2^	198	16.4 (14.1–19.0)	17.1 (14.9–19.3)	
**Mothers received antenatal care**				
No	68	18.4 (14.1–23.6)	18.2 (14.3–22.1)	0.0002
Yes	481	10.9 (9.9–12.1)	11.4 (10.4–12.3)	
**Mothers received postnatal care**				
No	137	8.8 (7.2–10.6)	8.9 (7.5–10.4)	0.0004
Yes	411	13.0 (11.6–14.4)	13.4 (12.2–14.6)	
**Mothers attitude toward wife-beating**				
Not justified	693	10.6 (9.6–11.7)	10.8 (10.1–11.6)	0.033
Justified	191	13.0 (10.9–15.3)	13.5 (11.7–15.3)	
**Mother's decision-making autonomy**				
Practiced	133	11.4 (9.5,13.6)	12.3 (10.3–14.2)	0.718
Not practiced	737	11.0 (10.0,12.1)	11.2 (10.4–11.9)	
**Number of living children**				
Less than 3 children	564	9.9 (9.0–11.0)	10.1 (9.3–10.9)	0.0001
3 children and above	320	13.9 (12.0–16.0)	14.3 (12.8–15.7)	
**Unwanted child**				
No	600	11.6 (10.5–12.7)	12.1 (11.2–13.0)	0.210
Yes	63	13.6 (10.6–17.3)	12.8 (9.8–15.7)	
**Place of delivery**				
Non-institutional	365	14.7 (13.0–16.6)	15.2 (13.8–16.6)	<0.001
Institutional	222	8.9 (7.6–10.4)	9.2 (8.1–10.4)	
**Ever had terminated pregnancy**				
No	727	11.1 (10.1–12.2)	11.4 (10.6–12.1)	0.716
Yes	157	10.8 (9.0–12.8)	11.2 (9.5–12.8)	
**Last birth a cesarean section**				
No	530	13.8 (12.4–15.3)	14.3 (13.2–15.4)	<0.001
Yes	132	7.4 (6.1–9.0)	7.6 (6.3–8.8)	
**Sign of Pregnancy complication**				
No	300	10.7 (9.4–12.2)	11.1 (10.0–12.3)	0.609
Yes	181	11.3 (9.7–13.1)	11.7 (10.1–13.3)	
**Children age**				
0–11 months	150	8.1 (6.7–9.7)	8.9 (7.5–10.2)	<0.001
12–23 months	213	12.6 (10.9–14.6)	13.3 (11.6–14.9)	
24–35 months	224	15.2 (13.1–17.4)	14.9 (13.1–16.7)	
36–47 months	147	10.2 (8.4–12.3)	10.1 (8.6–11.7)	
48–59 months	150	9.5 (7.7–11.5)	9.7 (8.2–11.1)	
**Sex of child**				
Male	470	11.2 (10.1–12.4)	11.6 (10.6–12.5)	0.753
Female	414	10.9 (9.6–12.4)	11.1 (10.1–12.1)	
**Birth order**				
First	293	9.8 (8.6–11.2)	9.8 (8.8–10.9)	<0.001
Second	269	10.1 (8.8–11.6)	10.6 (9.4–11.8)	
Third	158	11.5 (9.8–13.6)	12.0 (10.2–13.8)	
Fourth and above	164	16.7 (13.8–20.1)	16.9 (14.6–19.3)	
**Low birth weight**				
No	124	6.7 (5.5–8.2)	6.7 (5.6–7.9)	<0.001
Yes	63	18.9 (14.3–24.4)	19.1 (14.9–23.3)	
Not weighted	400	14.5 (12.9–16.3)	15.2 (13.8–16.5)	
**Currently had illness**				
No	460	11.0 (9.9–12.3)	11.3 (10.3–12.3)	0.865
Yes	424	11.1 (9.9–12.5)	11.4 (10.3–12.4)	
**Source of drinking water**				
Improved	780	11.3 (10.3–12.4)	11.5 (10.7–12.2)	0.107
Unimproved	104	9.4 (7.6–11.6)	10.3 (8.4–12.1)	
**Solid waste use in cooking**				
No	178	8.2 (6.8–9.8)	7.9 (6.8–9.0)	0.0001
Yes	703	12.2 (11.1–13.5)	12.7 (11.8–13.5)	
**Type of toilet facility**				
Improved	431	9.8 (8.7–11.0)	9.7 (8.8–10.6)	0.0006
Unimproved	453	12.7 (11.4–14.2)	13.5 (12.3–14.6)	
**Maas media exposure**				
No	415	14.5 (12.8–16.5)	14.7 (13.4–16.0)	<0.001
Yes	469	9.2 (8.2–10.2)	9.4 (8.6–10.2)	
**Wealth index**				
Poorest	288	16.2 (14.2–18.5)	16.6 (14.8–18.3)	<0.001
Poorer	221	13.1 (11.3–15.2)	14.0 (12.3–15.7)	
Middle	144	9.6 (7.8–11.7)	10.2 (8.6–11.8)	
Richer	141	9.4 (7.7–11.5)	9.1 (7.6–10.5)	
Richest	90	6.1 (4.8–7.7)	5.9 (4.7–7.1)	
**Place of residence**				
Urban	261	9.7 (8.1–11.6)	9.8 (8.7–10.9)	0.092
Rural	623	11.6 (10.4–12.8)	12.1 (11.2–13.0)	
Total	884	11.1 (10.1–12.1)	11.3 (10.6–12.0)	

a Regression-based prevalence.

CI, confidence interval.

### Factors associated with SAF

The most influential factors (based on Wald statistics) associated with under-5 SAF measured by the CISAF were children of age group 24–35 months (OR: 2.43, 95% CI: 1.83, 3.23, *p* < 0.001, Wald statistics: 6.13) (vs. youngest children), children born with low birth weight (OR: 3.33, 95% CI: 2.24, 4.97, *p* < 0.001, Wald statistics: 5.94) (vs. healthy weight children), children of underweight mothers (OR: 1.82, 95% CI: 1.44, 2.29, *p* < 0.001, Wald statistics: 5.09) (vs. healthy mothers), children of parents with no formal education (OR: 2.28, 95% CI: 1.56, 3.31, *p* < 0.001, Wald statistics: 4.31) (vs. children with educated parents), children from lower socio-economic quintile (OR: 2.25, 95% CI: 1.55, 3.26, *p* < 0.001, Wald statistics: 4.25) (vs. highest socio-economic quintile), children born by non-cesarean (OR: 1.56, 95% CI: 1.09, 2.22, *p* = 0.010, Wald statistics: 2.45) (vs. cesarean delivery) and children of mothers who did not receive antenatal care (OR: 1.54, 95% CI: 1.08, 2.20, *p* = 0.020, Wald statistics: 2.38) (vs. mothers received antenatal care) (Table 4).

**Table 4:** Factors associated with SAF among under-5 children.

**Characteristics**	**AOR (95% CI)**	***p* values**	**Wald statistics**	**ARD (95% CI)**	***p* values**
**Parents' education^a^^,^^b^**				
Both parents uneducated	2.28 (1.56–3.31)	<0.001	4.31	−0.09 (−0.16 to −0.02)	0.001
Only father uneducated	1.52 (1.16–1.99)	<0.001	3.06		
Only mother uneducated	1.64 (1.08–2.51)	0.020	2.31		
Both parents educated	1.00				
**Women working status^a^^,^^b^**				
Currently not working	1.14 (0.94–1.38)	0.180	1.33	−0.01 (−0.03–0.01)	0.410
Currently working	1.00				
**Mother's BMI^a^^,^^b^**				
≥18.5 kg/m^2^	1.00			0.09 (0.06–0.12)	<0.001
<18.5 kg/m^2^	1.82 (1.44–2.29)	<0.001	5.09		
**Mothers attitude toward wife–beating^a^^,^^b^**				
No	1.00			0.01 (−0.02–0.04)	0.432
Yes	1.04 (0.84–1.28)	0.710	0.37		
**Number of living children**				
Less than 3 children	1.00			0.01 (−0.03–0.06)	0.550
3 children and above	1.08 (0.73–1.59)	0.701	0.38		
**Mother received antenatal care^b^**				
No	1.54 (1.08–2.20)	0.020	2.38	−0.04 (−0.08–0.001)	0.040
Yes	1.00				
**Mother received postnatal care^b^**				
No	0.86 (0.65–1.14)	0.310	−1.01	0.01 (−0.02– 0.04)	0.631
Yes	1.00				
**Unwanted child**				
No	1.00			0.02 (−0.03–0.07)	0.490
Yes	1.25 (0.79–1.96)	0.340	0.96		
**Place of delivery**				
Non–institutional	1.00			−0.001 (−0.04–0.04)	0.991
Institutional	1.01 (0.68–1.49)	0.961	0.05		
**Last birth a cesarean section**				
No	1.56 (1.09–2.22)	0.010	2.45	−0.03 (−0.06–0.002)	0.082
Yes	1.00				
**Children age^a^^,^^b^**				
0–11 months	1.00			0.04 (0.01–0.07)	0.003
12–23 months	1.47 (1.13–1.91)	<0.001	2.87		
24–35 months	2.43 (1.83–3.23)	<0.001	6.13		
36–47 months	2.09 (1.57–2.78)	<0.001	5.10		
48–59 months	1.69 (1.27–2.26)	<0.001	3.59		
**Birth order^a^^,^^b^**				
First	1.00			0.01 (−0.03–0.03)	0.950
Second	1.10 (0.88–1.37)	0.390	0.86		
Third	1.03 (0.70–1.53)	0.871	0.17		
Fourth and above	1.33 (0.84–2.11)	0.221	1.22		
**Low birth weight^b^**				
No	1.00			0.19 (0.13–0.25)	<0.001
Yes	3.33 (2.24–4.97)	<0.001	5.94		
**Source of drinking water**				
Improved	1.00			−0.01 (−0.06–0.03)	0.511
Unimproved	0.84 (0.59–1.21)	0.360	−0.93		
**Solid waste use in cooking**				
No				0.01 (−0.03–0.05)	0.551
Yes	1.10 (0.80–1.51)	0.570	0.57		
**Type of toilet facility**				
Improved				0.02 (−0.01, 0.04)	0.210
Unimproved	1.16 (0.94–1.43)	0.161	1.43		
**Maas media exposure^a^^,^^b^**				
No	1.06 (0.91–1.24)	0.410	1.41	−0.01 (−0.04–0.01)	0.372
Yes	1.00				
**Wealth index^a^^,^^b^**				
Poorest	2.21 (1.47–3.33)	<0.001	3.80	−0.02 (−0.06–0.01)	0.230
Poorer	2.25 (1.55–3.26)	<0.001	4.25		
Middle	1.48 (1.04–2.12)	0.030	2.15		
Richer	1.89 (1.36–2.63)	<0.001	3.78		
Richest	1.00				
**Place of residence**				
Urban	1.13 (0.89–1.43)	0.311	1.02	0.02 (−0.01–0.04)	0.282
Rural	1.00				

CI, confidence interval; AOR, adjusted odds ratio; ARD, adjusted risk difference.

a Regression analysis adjusted for all variables except mother received antenatal care, mother received postnatal care, low birth weight,

b Regression analysis controlled for all variables.

Significant adjusted risk differences (24) between no SAF and SAF were observed for indicators, such as, parents' education (ARD = −0.09), underweight mother (ARD = 0.09), mother received antenatal care (ARD = −0.04), children's age (ARD = 0.04) and low birth weight (ARD = 0.19) (Table 4).

## Discussion

The prevalence of SAF among children under-5 in Bangladesh was 11.3%. In contrast, in the same population, the prevalence of severe stunting was 9% (16). Conventional indicators (i.e., severe stunting, severe wasting and severe underweight) can partly overlap, therefore may not provide a convincing estimate of the overall proportion of AF children in the population. On the other hand, CISAF uses conventional nutritional and growth indicators' aggregate values to estimate the overall proportion of SAF, thus provide a more convincing estimation of the overall proportion of AF children in the population (14). Previously no studies have widely addressed the overall proportion of SAF in Bangladesh based on the CISAF. Islam et al. recorded the prevalence of severe stunting (11.4%), severe wasting (3.1%) and severe underweight (7.7%) among children under-5 in Bangladesh using 2014 survey (25). In Nepal, Pravana et al. and Tiwari et al. revealed the prevalence of severe wasting (4.14%) and severe stunting (10.2%) respectively (26, 27). This study underlines that there are noticeable differences between the prevalence of SAF based on the CISAF and conventional disaggregated indicators in Bangladesh.

Most influential maternal characteristics associated with SAF were children of underweight mothers and children of parents with no formal education. Poor nutritional status of mothers is a potential risk factor of SAF (18, 25, 27). Mothers being underweight might affect the intrauterine growth of the fetus during the antenatal period, which in return affects the latter nutritional status of children (28). In addition, the poor nutritional status of women has been shown to be connected with poor distribution of inadequate food within the family along with food insecurity, poverty and micronutrient deficiencies that often tend to create macronutrient deficiencies in children (29). Again, parental education is another important associated factor of overall SAF has not been widely articulated apart from identifying association between parental educations and disaggregated conventional nutritional and growth indicators (i.e., stunting, wasting and underweight) (30, 31). Several studies have tried to introduce a link between mother's education and income by saying that increased schooling enables mothers to earn higher incomes and a similar argument can be made for paternal education that influences child's health (32, 33). However, it is difficult to assess the relative importance of income as a mediator for parental education effects. A comprehensive child SAF prevention package should include comprehensive health education and the use of nutritional interventions to enhance overall physical health and nutritional status of mothers and children (34).

Children of age group 24–35 months and born with low birth weight were more likely to suffer SAF. However, children born with low birthweight was previously identified as key factor associated with severe stunting in some developing countries like Pakistan, Nepal, Malawi, Mexico and Iran; Children of age group 24–35 months found insignificant in Nepal and Nigeria (18, 26, 35–38). After the 2nd year of life, children in Bangladesh tend to have the same diet as the family and only have access to inadequate amounts of solid food that can lead to a poor nutritional and growth status (39). Parents should offer healthy foods to the children age >2 years to avoid SAF. Exclusive breastfeeding is still not satisfactory in Bangladesh and this might have minimized the risk of AF in future (16). On the other hand, children with low birth weight experience faltered growth during early childhood that can lead to an increased risk of complications in later life (40). Children with synchronized conditions of AF and low birth weight have an increased susceptibility to infection such as diarrhea plus lower respiratory infections, sleep apnea, jaundice, anemia, chronic lung disorders, fatigue and loss of appetite (4). Appropriate initiation of complementary feeding might reduce the risk of SAF in later stage (41).

The odds of being SAF increased among children from less wealthy households, as also evidenced by various previous studies (6, 25, 42, 43). One fifth of the children living in socio-economically poorest households experienced SAF. Parents of having such status often cannot afford minimum diet and quality health care for their children (28, 44).

The findings indicate that Bangladesh has not yet achieved sustainable improvement in child nutrition and growth. Concerted efforts to enable strong collaboration among multiple sectors such as government, non-government, social, cultural, and religious institutions in order to improve nutritional and growth status are essential, particularly to address the severe adverse nutritional and growth outcomes. SAF can be reduced by taking measures to strengthen action around social determinants of health, such as, increasing the participation of women and girls in education, reducing socio-economic inequality by enhancing income generating sectors and establishing specific health and nutrition programmes. Further, empirical education and quality health care should be made available and accessible to all women will confer many benefits, for example, improve caregiver practices, enhance health and environmental knowledge; increase educated and skill work force; to live in better neighborhoods, reduce gender-based violence; reduces child marriage and early childbearing; reduces maternal death rates for the betterment of nutritional status and child development (45).

This study has several strengths. Firstly, a wide range of parental, household and child characteristics have been used to explain the SAF based on the CISAF among children under-5 living in Bangladesh. Secondly, the sample size is representative of that country and, based on the complex nature of the data, sophisticated statistical analyses have been used in order to meet the main objective of the study. There were limitations, however. For example, the cross-sectional nature of the study means that it was not possible to establish a causal relationship between risk factors and SAF. Diet practice, ethnicity and parental behavioral factors were not controlled in this study due to the unavailability of a large amount of necessary information covering these areas. Another limitation is recall bias or information bias that can occur from information collected by self-reporting such as age, education, occupation, and household assets etc. Overall, CISAF used seven measurements of nutritional and growth status provided a credible estimate of the overall proportion of under-5 SAF and the complex interplay between individual, community, public policy and environment level risk factors. Although the composite index is a more convincing estimate to represent the overall burden of SAF among under-5 children, it is not popular among pediatricians and public health researchers. Because this method has not been widely studied and validated, more research in this area is recommended.

## Conclusion

The overall prevalence of under-5 child SAF was greater than conventional nutritional and growth indicators. One in 10 children experiences SAF in Bangladesh. Children born with low birth weight, those living in socio-economically poorest households, children of age group 24–35 months, children of underweight mothers and children in fourth and above birth order are important key factors associated with SAF. Prioritizing and ensuring context-specific interventions addressing individual, community, public policy and environment level risk factors from policy level to implementation to reduce structural and intermediary determinants of under-5 SAF.

## Data availability statement

The datasets presented in this study can be found in online repositories. The names of the repository/repositories and accession number(s) can be found at: https://dhsprogram.com/data/available-datasets.cfm.

## Ethics statement

Ethical approval for using the data was obtained from ICF International Rockville, Maryland, USA. Since the data was collected from secondary sources, ethical approval and written consent for participation were not required in this research.

## Author contributions

MC designed the study plan. MC and HK performed statistical analyses. MC and FB prepared figures and tables. MC, FB, and MR drafted the manuscript. MM and BB edited it. All authors approved the study and the final manuscript.

## Conflict of interest

The authors declare that the research was conducted in the absence of any commercial or financial relationships that could be construed as a potential conflict of interest.

## Publisher's note

All claims expressed in this article are solely those of the authors and do not necessarily represent those of their affiliated organizations, or those of the publisher, the editors and the reviewers. Any product that may be evaluated in this article, or claim that may be made by its manufacturer, is not guaranteed or endorsed by the publisher.
