# Supplementary material for: Prevalence and correlates of severe under-5 child anthropometric failure measured by the composite index of severe anthropometric failure in Bangladesh
Source: Front Pediatr. 2022 Sep 14;10:978568. doi: 10.3389/fped.2022.978568 (PMC9516305; doi:10.3389/fped.2022.978568)

Supplementary Table 1 | Measurements of independent variables

Variable	Description	Measurement	Scale of measurement
**Mothers' age (in years)**	Age of mothers at the time of data collection	15-19, 20-24, 25-29, 30-34, 35-39, ≥ 40	Discrete, categorial
**Parents' education**	Parental educational status. Accomplishment of at least five years of schooling (primary level, class 1 to 5) refers educated. No (0 years of schooling) schooling year refers uneducated.	Both parents were uneducated, only father was uneducated, only mother was uneducated, both parents were educated	Categorical
**Mother's income-earning status**	Mother engaged in economic activity at the time of data collection	Not working, Currently working	Binary
**Underweight mother**	Mother with <18.5 kg/m^2 of body mass index refers to underweight, and ≥18.5 kg/m^2 refers to healthy or not underweight	No, Yes	Binary
**Mothers received antenatal care**	At least one medical surveillance and review performed during pregnancy for the early detection of possible complications of pregnancy	No, Yes	Binary
**Mothers received postnatal care**	At least one postnatal care is the individualized care provided to meet the needs of a mother and her baby following childbirth	No, Yes	Binary
**Mother's attitudes toward wife-beating**	Respondents are asked if they agree that a husband is justified in hitting or beating his wife under each of the following five circumstances: she burns the food, she argues with him, she goes out without telling him, she neglects the children, and she refuses to have sex with him. If respondents answer "yes" in at least one circumstance, they are considered to have attitudes justifying wife beating.	Not justified, Justified	Binary
**Mothers' decision-making autonomy**	In the BDHS surveys, a woman's decision-making power is assessed on the following three themes: 1) a woman who usually decides on her healthcare 2) a woman who usually decides on large household purchases and 3) a woman who usually decides on visits to family or relatives. The response options were as follows: (a) respondent alone, (b) respondent and husband/partner, (c) respondent and another person, (d) husband/partner alone, (e) someone else, (f) other. For each question, a value of 1 was assigned for inability in decision-making if the responses were d, e, or f	Not participated, Participated	Binary

	and 0 for otherwise if the responses were a, b, or c. The values were then added, resulting in a score from 0 to 3. The Cronbach's α for the instruments was 0·79, indicating high internal consistency.		
**Number of living children**	Mothers gave live birth and they were live during the interview.	Less than 3 children, 3 children and above	Binary
**Unwanted child**	Mothers did not want to get pregnant with the current baby.	No, Yes	Binary
**Place of delivery**	Children born in public or private health facilities is referred to as institutional delivery whereas giving birth at home denotes non-institutional delivery.	Non-institutional, institutional	Binary
**Ever had terminated pregnancy**	Women had miscarriages, abortions or still birth	No, Yes	Binary
**Last birth a caesarean section**	Women had caesarean section before the last birth.	No, Yes	Binary
**Sign of Pregnancy complication**	Mothers reported Pregnancy complication during antenatal visits	No, Yes	Binary
**Children's age (in months)**	Age of the children at the time of data collection	0-11 months, 12-23 months, 24-35 months, 36-47 months, 48-59 months	Categorical
**Sex of child**	Sex differential of children	Male, Female	Binary
**Birth order**	Birth order is the chronological order of sibling births in a family	One, two, three, four and above	Categorical
**Low birth weight**	Children were <2.5 kg of weight during birth. Approximately 75% mothers can correctly report their baby's size at birth, therefore mother's recall is a valid proxy measure of birth weight.	No, Yes, Not weighted	Categorical
**Currently had illness**	Children had at least cough, fever or diarrhea before 2 weeks of the survey	No, Yes	Binary
**Source of drinking water**	Improved: piped into dwelling, piped to yard/plot, public tap/standpipe, piped to neighbour, tube well or borehole, protected well, protected spring, rainwater, tanker truck, cart with small tank, bottled water; unimproved: unprotected well, unprotected spring, surface water (river, dam, lake, pond, stream, canal, irrigation channel), other	Improved, unimproved	Binary
**Type of toilet facility**	Improved: flush - to piped sewer system, flush - to septic tank, flush - to pit latrine, flush - don't know where, pit latrine - ventilated improved pit, pit latrine - with slab, composting toilet; unimproved: flush - to somewhere else, pit latrine - without slab / open	Improved, unimproved	Binary

	pit, bucket toilet, hanging toilet/latrine, other		
**Solid waste used in cooking**	Solid fuel includes coal, lignite, charcoal, wood, straw / shrubs / grass, agricultural crop and animal dung; Clean fuel includes electricity, natural gas, processed gas, biogas, kerosene	Clean fuel, Solid fuel	Binary
**Mass media exposure**	Mass media exposure through television, radio and newspaper/magazine has been defined as exposure to at least one media that exposes to at least once a week	No, Yes	Binary
**Wealth index**	Wealth index in the DHS surveys is calculated, by the DHS authority, based on information on household characteristics and assets using principal component analysis. Then households are classified into quintiles based on the values of the wealth index, where households with lower values of the index is considered as poorest and vice-versa	Poorest, poorer, middle, richer, richest	Categorical
**Place of residence**	Women’s place of residence	Urban, rural	Binary

Supplementary Figure 1 | Prevalence of severe anthropometric failure

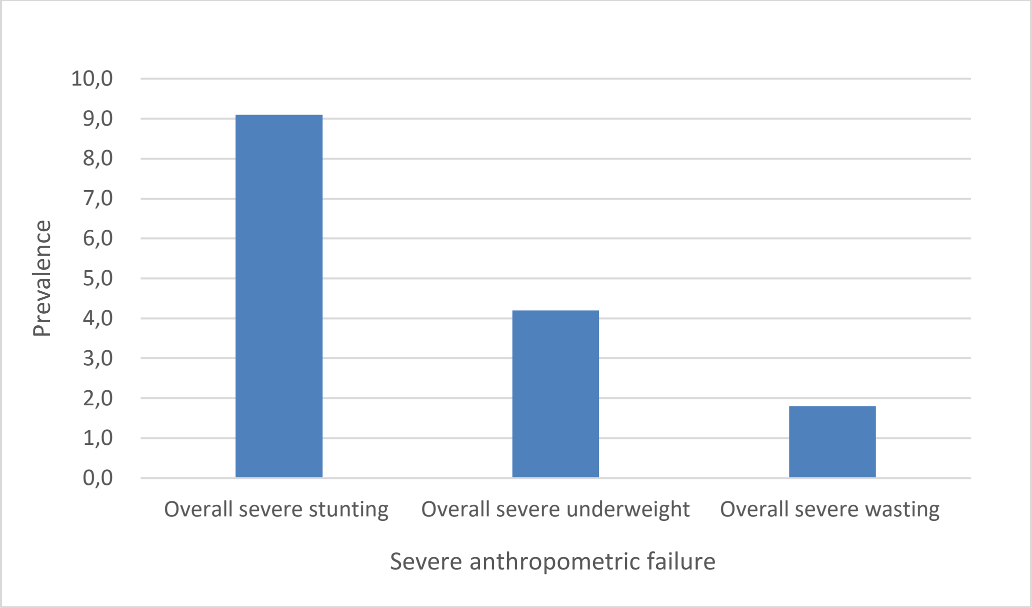

Supplement: Supplementary file 1 [file Data_Sheet_1.PDF]
